# The relationship between hysterectomy, menopause, and tubal ligation, with coronary heart diseases in North of Iran: a population-based case–control study

**DOI:** 10.1038/s41598-025-03480-z

**Published:** 2025-05-29

**Authors:** Mehran Asadi-Aliabadi, Mahmood Moosazadeh, Kiarash Shakeriastani, Mohammadmehdi Pejman, Mobina Gheibi, Maliheh Ghasemi Tirtashi, Erfan Ghadirzadeh

**Affiliations:** 1 Health Sciences Research Center, Mazandaran University of Medical Sciences, Sari, Iran; 2 Gastrointestitional Cancer Research Center, Non-Communicable Disease Institute, Mazandaran University of Medical Sciences, P.O.BOX: 4816117949, Sari, Iran; 3 Student Research Committee, School of Medicine, Mazandaran University of Medical Sciences, Sari, Iran; 4 Student Research Committee, Kerman University of Medical Sciences, Kerman, Iran; 5 Department of Obstetrics and Gynecology, Faculty of Medicine, Mazandaran University of Medical Sciences, Sari, Iran; 6 Student Research Committee, School of Allied Medical Sciences, Mazandaran University of Medical Sciences, Sari, Iran

**Keywords:** Coronary heart disease, Hysterectomy, Menopause, Tubal ligation, Risk factor, Cohort, Cardiology, Risk factors, Cardiovascular diseases, Reproductive disorders, Disease prevention, Public health

## Abstract

Previously, menopause, hysterectomy, and tubal ligation (TL) have been evaluated as coronary heart disease (CHD) risk factors. However, the results regarding the significance of these associations were conflicting. Thus, the present study aimed to assess whether hysterectomy, menopause, and TL increase the odds of CHD. This case-control study included data from the enrollment phase of the Tabari cohort study (TCS) consisting of 564 cases of CHD and 564 healthy controls. Logistic regression was used to calculate the odds ratio (OR) of CHD in relation to hysterectomy, menopause, and TL status after adjustment for confounders. The univariate logistic regression analysis showed a significantly higher odds of CHD among post-menopausal participants (OR: 5.09, 95%CI 3.92–6.61), participants with TL (OR: 1.81, 95%CI 1.41–2.32), and women with hysterectomy (OR: 2.43, 95%CI 1.69–3.50). However, none of the associations were statistically significant (Hysterectomy: OR: 1.21, 95%CI 0.8–1.85; Menopause: OR: 1.43, 95%CI 0.88–2.31; TL: OR: 1.01, 95%CI 0.74–1.37) in the fully adjusted model (after adjustment for age, diabetes, hypertension, residential area, waist-to-hip ratio, pregnancy number, socio-economic state, occupation, education, and physical activity). Although some models showed significance, none of the reproductive factors showed a significant association with CHD after full adjustment.

## Introduction

Coronary heart diseases (CHD) represent as the leading cause of morbidity and mortality worldwide, especially among developing countries^1^. The multifactorial etiology of CHD encompasses genetic predispositions, lifestyle risk factors, and medical conditions and procedures^2,3^. In women, emerging evidence suggests that reproductive factors, such as pregnancy, hysterectomy, menopause, and tubal ligation (TL), may further modulate CHD risk, though findings remain inconclusive^4–6^.

Hysterectomy is the surgical removal of the uterus and one of the most common gynecological procedures worldwide^7^. While several studies associate hysterectomy with increased cardiovascular risk, the magnitude and significance of this relationship vary substantially across populations. For instance, Farland et al.^8^ demonstrated that hysterectomy was associated with a 19% increased odds of CVD among U.S. females, whereas Wang et al.^9^ reported a more pronounced 45% higher odds of CHD in their study. Although both studies suggested hysterectomy as a factor contributing to CVDs, the effect sizes differed.

These discrepancies may stem from differences in study design, sample characteristics (e.g., age, ethnicity), or adjustment for confounders such as oophorectomy status and hormone therapy. On the other hand, Choi et al.^10^ and Yeh et al.^11^ found no relationship between hysterectomy and CHD, highlighting the ongoing debate regarding its cardiovascular implications. Such inconsistencies underscore the need for further investigation, particularly in understudied populations like those in the Middle East.

Previous research has also assessed the relationship between other female reproductive factors, such as menopause, and CHD with conflicting results. Agrinier et al.^12^ found that higher Framingham 10-year risk of CHD was more prevalent among postmenopausal women than premenopausal females. In contrast, Colditz et al.^13^ observed no independent effect of menopause on CHD. Notably, many prior studies have either focused broadly on cardiovascular diseases (CVD) rather than CHD specifically or examined only intermediate outcomes (e.g., lipid profiles and anthropometric indices), leaving gaps in clinical endpoint data. Also, TL, another common gynecological procedure, has been poorly investigated in relation to CHD, with limited evidence.

The discrepancies among the results of previous studies as well as the limited knowledge in this field, require extensive research. Thus, given these unresolved questions and regional gaps in evidence, the present study was conducted with two major goals in a large population of northern Iranians: First, to assess whether hysterectomy, menopause, and TL are associated with higher odds of CHD, and second, to evaluate potential determinants (including demographic and cardiometabolic profile) contributing to CHD.

## Methods

### Study design and population

This case-control study was conducted on the enrollment phase data of the TABARI cohort study (TCS). TCS is a subset of the “Prospective Epidemiological Research Studies in Iran (PERSIAN)” which is a larger Iranian national cohort. In the enrollment phase of the TCS, 10,255 participants aged 35 to 70 years old were enrolled between 2015 and 2017. Using a census-based sampling method, participants residing in Sari, Mazandaran, Iran were enrolled in the TCS.

### Inclusion and exclusion criteria

Among 6106 female participants of the TCS, 564 individuals with self-reported history of CHD (including stable angina pectoris, unstable angina, myocardial infarction, percutaneous coronary intervention, or coronary artery bypass graft) were included as the case group, and 564 healthy individuals were included as the control group. Researchers checked participants medical records and relevant documentation to verify their medical history; thus, reducing the possibility of recall bias. Participants with kidney failure and a history of cancer were excluded.

### Cohort data collection tools and methods

Trained researchers utilized standardized questionnaires to collect data regarding demographic information and the medical history of the participants as per the PERSIAN cohort protocol. Data curation approach combined assessment of self-reported data alongside medical records and validated questionnaires by healthcare professional staff. Cohort participants were required to provide detailed health documentation and medical records during the enrollment phase.

The Cohort survey includes 482 questions grouped into three main categories: general information, health-related details, and dietary habits. Each section is conducted by a skilled interviewer. Since there is no fixed sequence for answering the questions, participants are instructed to fill out the sections depending on which interviewer is available at the time.

Please refer to the PERSIAN cohort and TCS methodology papers for further details on the data collection techniques used^14–16^.

### Measurements

Demographic data and medical history of the participants including age, residential area (urban or mountainous), marital status (single, married, widowed, or divorced), socio-economic status (from 1 as the lowest to 5 as the highest), occupation, education (years of schooling, or having a college or university degree), physical activity (PA) level (measured in metabolic equivalent (METs) of below or above median), history of CHD, diabetes (DM), dyslipidemia (DLP), history of hypertension (HTN), and female reproductive factors (including pregnancy, age at menarche (AAM) categorized into three groups based on their AAM: early menarche (at age 11 or younger), normative menarche (at age 12–13), or late menarche (at age ≥ 14), hysterectomy, menopause, and TL) were extracted from the TCS data repository system which were gathered by general and medical parts of the cohort questionnaire. Anthropometric measures, including body mass index (BMI), waist circumference (WC), measured with a non-elastic tape from the midpoint between the iliac crest and the lower rib margin, and waist-to-hip ratio (WHR), were also extracted from the TCS data repository system which were measured using SECA 226 stadiometer and SECA 755 analogue standing scale (SECA, Hamburg, Germany) in the morning to the nearest 0.1 cm and kg.

### Statistics

Data was analyzed using SPSS software version 26 (IBM SPSS Corp, USA). Descriptive statistics are reported using numbers and percentages. Univariate logistic regression was used to compare variables between cases and controls and to add variables with a P-value of less than 0.250 to the multivariate model as potential confounders. Stepwise multiple logistic regression was used to calculate the odds ratio (OR) and 95% confidence intervals (CI) of CHD in relation to hysterectomy, menopause, and TL status after adjustment for confounders (age, DM, HTN, residential place, WHR, pregnancy number, socio-economic level, education level, MET, and occupation) considering participants without these reproductive factors as the reference group. A P-value less than 0.05 was considered statistically significant.

### Ethics

TCS was approved by the Mazandaran University of Medical Sciences Ethical Committee (Ethics Approval Code: IR.MAZUMS.REC.95.2524). Written informed consent was obtained from all participants before entering the study. All procedures performed in this study were in accordance with the ethical standards of the Institutional Research Ethics Committee of Mazandaran University of Medical Sciences and with the 1964 Helsinki Declaration and its later amendments.

## Results

### Characteristics of cases and controls

Out of the 1128 women included in the study, one control was selected for each case, resulting in 564 women with CHD being included in the case group and 564 women without CHD being included in the control group. 102 (24.5%) cases and 314 (75.5%) controls aged below 50 years old while 228 (58.3%) cases and 163 (41.7%) controls aged between 50 and 59 years old, and 234 (72.9%) cases and 87 (27.1%) controls aged between 60 and 70. Additionally, 294 (43.4%) cases and 383 (56.6%) controls lived in urban areas, 182 (60.5%) cases and 119 (39.5%) controls were in the lowest socio-economic level, 214 (70.4%) cases and 90 (29.6%) control had no schooling. 437 (49.7%) cases and 442 (50.3%) controls had early menarche (age at menarche ≤ 11), 84 (50.3%) cases and 83 (49.7%) controls had late menarche (age at menarche ≥ 16), 238 (59.5%) cases and 162 (40.5%) controls had TL, 441 (65.4%) cases and 233 (34.6%) controls had reached menopause, and 104 (68.4%) cases and 48 (31.6%) controls had a history of hysterectomy. Table 1 shows further information regarding the baseline characteristics of cases and controls.

**Table 1:** Baseline characteristics of cases and controls.

Variables		Number of participant	Case (With CHD)		Control (Without CHD)		Univariate logistic regression	
			n	%	n	%	OR (95% CI)	*P*-value
Age	< 50	416	102	24.5	314	75.5	Ref	Ref.
	50–59	391	228	58.3	163	41.7	4.31 (3.19–5.82)	< 0.001
	60–70	321	234	72.9	87	27.1	8.28 (5.93–11.54)	< 0.001
Area residence	Urban	677	294	43.4	383	56.6	Ref	Ref.
	Mountainous	451	270	59.9	181	40.1	1.94 (1.53–2.48)	< 0.001
Social economic level	1 (lowest)	301	182	60.5	119	39.5	Ref	Ref.
	2	242	126	52.1	116	47.9	0.71 (0.50-1.00)	0.050
	3	217	110	50.7	107	49.3	0.67 (0.47–0.96)	0.027
	4	192	81	42.2	111	57.8	0.48 (0.33–0.69)	< 0.001
	5 (highest)	176	65	36.9	111	63.1	0.38 (0.26–0.56)	< 0.001
Education level	University/College	166	45	27.1	121	72.9	Ref	Ref.
	9–12 years in school	227	66	29.1	161	70.9	1.10 (0.70–1.72)	0.669
	6–8 years in school	105	54	51.4	51	48.6	2.85 (1.70–4.76)	< 0.001
	1–5 years in school	326	185	56.7	141	43.3	3.53 (2.35–5.30)	< 0.001
	No schooling	304	214	70.4	90	29.6	6.41 (4.19–9.75)	< 0.001
Has job	No	968	512	52.9	456	47.1	Ref	Ref.
	Yes	160	52	32.5	108	67.5	0.43 (0.30–0.61)	< 0.001
Marital status	Single/widow	152	79	52	73	48.0	Ref	Ref.
	marriage	976	485	49.7	491	50.3	0.91 (0.65–1.29)	0.601
AAM	≤ 11	879	437	49.7	442	50.3	1.11 (0.71–1.76)	0.637
	12–15	82	43	52.4	39	47.6	Ref	Ref.
	≥ 16	167	84	50.3	83	49.7	1.02 (0.73–1.42)	0.890
PA	< median	562	268	47.7	294	52.3	Ref	Ref.
	≥ median	566	296	52.3	270	47.7	1.21 (0.95–1.52)	0.122
Number of pregnancy	0 or 1	102	19	18.6	83	81.4	Ref	Ref.
	2	189	53	28	136	72.0	1.71 (0.94–3.07)	0.078
	3	212	96	45.3	116	54.7	3.61 (2.05–6.37)	< 0.001
	4	191	89	46.6	102	53.4	3.81 (2.15–6.77)	< 0.001
	≥ 5	434	307	70.7	127	29.3	10.56 (6.16–18.11)	< 0.001
TL	No	728	326	44.8	402	55.2	Ref	Ref.
	Yes	400	238	59.5	162	40.5	1.81 (1.41–2.32)	< 0.001
Menopause	No	454	123	27.1	331	72.9	Ref	Ref.
	Yes	674	441	65.4	233	34.6	5.09 (3.92–6.61)	< 0.001
Hysterectomy	No	976	460	47.1	516	52.9	Ref	Ref.
	Yes	152	104	68.4	48	31.6	2.43 (1.69–3.50)	< 0.001

TL, Tubal ligation; CHD, coronary heart disease; PA, physical activity; AAM, age at menarche; OR, odds ratio; CI, confidence interval.

### CHD demographic risk factors

With increasing age, a significant association was seen in the occurrence of CHD. Compared to women aged below 50 years, those aged 50–59 years and 60–70 years had significantly higher odds of having CHD (OR: 4.31, 95%CI: 3.19–5.82, and OR: 8.28, 95%CI: 5.93–11.54, respectively). In relation to the place of living, women living in mountainous areas had 1.94 times higher odds of having CHD compared to those living in urban areas (95%CI: 1.53–2.48). With the improvement of the socioeconomic level, the chance of developing CHD increased significantly. Compared to those in the lowest socio-economic level (level-1), individuals in the highest socio-economic (level-5) had a 62% (95%CI: 44-74%) reduced chance of CHD. Our results showed that as the level of education increases, the chance of CHD decreases. Compared to university/college graduates, participants with no schooling had 6.41 (95%CI: 4.19–9.75) times higher odds of having CHD. Women without a job had 2.33 (95%CI: 1.64–3.31) times higher odds of having CHD compared to those employed. Compared to women with 0–1 pregnancies, those with ≥ 5 pregnancies had 10.56 (95%CI: 6.16–18.11) times higher odds of having CHD. Women with TL, menopausal women, and women with a history of hysterectomy had 1.81 (95%CI: 1.41–2.32), 5.09 (95%CI: 3.92–6.61), and 2.43 (95%CI: 1.69–3.50) times higher odds of CHD, respectively. On the other hand, marital status, age at menarche, and PA level, presented as metabolic equivalent (METs), had no significant association with CHD (Table 1).

### CHD and cardiometabolic profile

Table 2 shows the results of the association between metabolic risk factors and CHD. DLP was not significantly associated with CHD (OR: 1.01, 95%CI: 0.77–1.33). However, compared to women with normal BMI, the obese women (BMI ≥ 30) had a significantly higher odds of CHD (OR: 1.55, 95%CI: 1.11–2.17). Those with WC ≥ 88 cm had a 1.94 (95%CI: 1.48–2.54) fold higher odss of CHD compared to those with WC < 88 cm. Those with WHR > 0.85 had a 2.63 (95%CI: 1.94–3.56) times higher odds of CHD compared to those with WHR ≤ 0.85. Also, DM and HTN were associated with 2.61 times (95%CI: 1.98–3.45) and 4.52 times (95%CI: 3.50–5.86) higher odds of CHD.

**Table 2:** Cardiometabolic profile of cases and controls.

Variables		Number of participant	Case (With CHD)		Control (Without CHD)		Univariate logistic regression	
			n	%	n	%	OR (95% CI)	*P*-value
DLP	No	263	131	49.8	132	50.2	Ref	Ref.
	Yes	865	433	50.1	432	49.9	1.01 (0.77–1.33)	0.944
BMI	< 25	191	83	43.5	108	56.5	Ref	Ref.
	25-29.9	426	203	47.7	223	52.3	1.18 (0.84–1.67)	0.334
	≥ 30	511	278	54.4	233	45.6	1.55 (1.11–2.17)	0.010
WC	< 88	307	117	38.1	190	61.9	Ref	Ref.
	≥ 88	821	447	54.4	374	45.6	1.94 (1.48–2.54)	< 0.001
WHR	≤ 0.85	240	76	31.7	164	68.3	Ref	Ref.
	> 0.85	888	488	55	400	45.0	2.63 (1.94–3.56)	< 0.001
DM	No	830	364	43.9	466	56.1	Ref	Ref.
	Yes	298	200	67.1	98	32.9	2.61 (1.98–3.45)	< 0.001
HTN	No	693	251	36.2	442	63.8	Ref	Ref.
	Yes	435	313	72	122	28.0	4.52 (3.50–5.86)	< 0.001

CHD, coronary heart disease; DLP, dyslipidemia; BMI, Body mass index; WC, waist circumference; WHR, waist-to-hip ratio; DM, diabetes mellitus; HTN, hypertension; OR, odds ratio; CI, confidence interval.

### Association of hysterectomy, TL, and menopause with CHD

Table 3 shows the association between reproductive factors including menopause, TL, and hysterectomy, with odds of CHD. The analysis was adjusted for diverse sets of confounders through nine models. In model-1 (adjusted only by age), the odds of CHD was 1.81 times higher in menopausal patients (OR: 1.81, 95%CI: 1.17–2.79), and 1.37 times higher in women who underwent TL (OR: 1.37, 95%CI: 1.05–1.80). In model-2 (adjusted by age, DM, and HTN) the odds of CVDs was 1.61 fold in menopausal women compared to non-menopausal (OR: 1.61, 95%CI: 1.03–2.53), and 1.35 times higher in women who underwent TL than who did not (OR: 1.35, 95%CI: 1.02–1.78). In Model-3 (adjusted by age, DM, HTN, and residential place), the odds of CHD was only significantly higher in menopausal patients compared to non-menopausal patients (OR: 1.59, 95%CI: 1.01–2.50) whereas hysterectomy and TL showed non-significant associations. Finally, the full Models (Models 7–9: Adjusted by age, DM, HTN, residential place, WHR, pregnancy number, socio-economic level, education level, MET, occupation, and two of the hysterectomy, menopause, or TL) did not show any significant associations between higher odds of CHD and these reproductive factors.

**Table 3:** Multiple logistic regression analysis of the association between hysterectomy, menopause, and TL with CHD risk (Participants without history of the mentioned reproductive factors were indicated as the reference group).

Variables		Multiple logistic regression	
		OR (95% CI)	*P*-value
Model-1	Hysterectomy	1.45 (0.98–2.13)	0.062
	Menopause	1.81 (1.17–2.79)	0.007
	Tubal ligation	1.37 (1.05–1.80)	0.022
Model-2	Hysterectomy	1.31 (0.88–1.96)	0.181
	Menopause	1.61 (1.03–2.53)	0.037
	Tubal ligation	1.35 (1.02–1.78)	0.037
Model-3	Hysterectomy	1.40 (0.94–2.10)	0.101
	Menopause	1.59 (1.01–2.50)	0.045
	Tubal ligation	1.29 (0.97–1.71)	0.076
Model-4	Hysterectomy	1.40 (0.94–2.10)	0.102
	Menopause	1.55 (0.98–2.44)	0.058
	Tubal ligation	1.27 (0.96–1.68)	0.097
Model-5	Hysterectomy	1.31 (0.87–1.97)	0.190
	Menopause	1.52 (0.96–2.40)	0.075
	Tubal ligation	1.05 (0.78–1.41)	0.763
Model-6	Hysterectomy	1.32 (0.87–1.99)	0.187
	Menopause	1.50 (0.95–2.40)	0.083
	Tubal ligation	1.02 (0.75–1.38)	0.901
Model-7	Hysterectomy	1.21 (0.80–1.85)	0.368
Model-8	Menopause	1.43 (0.88–2.31)	0.144
Model-9	Tubal ligation	1.01 (0.74–1.37)	0.956

Participants without history of these reproductive factors were indicated as the reference group. Model-1: Adjusted by age, Model-2: Adjusted by age, DM, and HTN, Model-3: Adjusted by age, DM, HTN, and residential place, Model-4: Adjusted by age, DM, HTN, residential place, and WHR, Model-5: Adjusted by age, DM, HTN, residential place, and WHR, and number of pregnancies, Model-6: Adjusted by age, DM, HTN, place, WHR, Pregnancy number, Social economic, Education level, MET, and occupation, Model-7: Adjusted by age, DM, HTN, residential place, WHR, pregnancy number, socio-economic level, education level, MET, occupation, menopause, and TL, Model-8: Adjusted by age, DM, HTN, residential place, WHR, pregnancy number, socio-economic level, education level, MET, occupation, hysterectomy, and TL, and Model-9: Adjusted by age, DM, HTN, residential place, WHR, pregnancy number, socio-economic level, education level, MET, occupation, hysterectomy, and menopause.

## Discussion

This study investigated the characteristics and determinants associated with higher odds of CHD in a cohort of 1128 women with a special perspective on women’s reproductive health (hysterectomy, menopause, and TL). Our findings showed that although women with hysterectomy, TL, and postmenopausal women are more common among the CHD group, the multiple logistic regression models suggested that hysterectomy, menopause, and TL are not significant determinants of CHD.

### Demographic characteristics and CHD

The results showed that a significant proportion of CHD patients were over 50 years old. This is consistent with other studies that have also proven the positive effect of increasing age on the occurrence of CHD^17,18^. Living in mountainous areas was identified as a determinant. Conversely, other studies showed that the increased odds of CHD is associated with living in urban areas, which could be attributed to factors such as air pollution and a sedentary lifestyle in urban areas^19,20^. One possible explanation for the discrepancy in our findings and the previous studies could be the age difference between the populations as a population residing in mountainous areas tends to be older compared to those living in urban areas which the analysis did not account for this factor. The education level played a crucial role, with a higher odds of CHD observed in individuals with lower educational attainment. Previous studies also showed an association between low education levels and increased odds of CVD^21–23^. Therefore, a higher level of education can make it possible for people to better understand and use public health services and preventive CHD recommendations, and lead to the improvement of health behaviors^23,24^. Unemployment was prevalent among CHD patients in our study, highlighting a potential link between compensation, socio-economic status, and disease risk. Other studies have also shown an association between unemployment and higher odds of CHD due to factors such as depression, stress, and reduced access to health care^25,26^.

### CHD and cardiometabolic factors

Metabolic risk factors such as obesity, DLP, DM, and HTN were more prevalent among CHD patients. In the present study, obesity, high WC, elevated WHR, DM, and HTN were significantly associated with an increased odds of CHD, underscoring the impact of metabolic health on cardiovascular outcomes. Consistently, previous studies have also shown the importance of obesity^27^, DLP^28^, DM^29^, HTN^30^, and WHR^31^ to developing CHD. However, in the present study, no difference was observed between the case and the control group in terms of DLP. This may be due to the higher prevalence of DLP among the overall Iranian population compared to other ethnicities even in normal participants. A meta-analysis by Darroudi et al.^32^ showed that the prevalence of DLP among Iranian adults was 83.4% while this number was 13.17% in China and 24.3% in Spain^33,34^. Additionally, most of our participants aged above 50 years which may justify our results since previous studies have shown that higher age can be a risk factor for DLP^35,36^.

### CHD and reproductive factors

Women with a history of hysterectomy, TL, and post-menopausal women showed significant associations with CHD in the Univariate analysis. However, the multiple logistic regression analysis, after adjustment for confounders, showed no significant relationship.

Our analysis did not reveal any significant association between hysterectomy and CHD in any of the models. This finding can be attributed to the confounding influence of age, as most hysterectomies were performed at older ages, which are often characterized by hormonal changes. A study on 2094 women showed that women who underwent hysterectomy at the age of 50 or older did not exhibit a heightened risk of CVD or metabolic conditions^37^.

Similar to our findings, Yeh et al.^11^ followed up 7605 females with a history of hysterectomy and 30,420 controls for 7 years in Taiwan. They found that there was no overall relationship between hysterectomy and CHD. The association was only significant in women who had hysterectomy prior to 45 years old. Also, a meta-analysis by Chen et al.^38^ showed that CHD risk was only higher in patients with both hysterectomy and oophorectomy and not patients with hysterectomy alone. In the present study, we could not conduct this subgroup analysis since we did not have the data regarding oophorectomy status in the TCS data registry. Consistently, Choi et al.^10^ studied 8642 Korean women with hysterectomy and 34,568 controls and found that hysterectomy was not a determinant for CHD and their association was only significant in participants below the age of 50.

On the other hand, inconsistent with our results, previous studies showed that women with a history of hysterectomy and/or TL either with or without ovary removal, because of rapid decline in the sex hormones such as estrogen and progesterone, were at a higher risk of developing CHD^8^. For instance, Wang et al.^9^ carried out a study on 15,257 US adult females and found that women with hysterectomy are at higher risk of CHD (OR: 1.45). This discrepancy may be due to differences in our statistical analysis methodology. Additionally, some studies also investigated the association between hysterectomy and CVD with most of them concluding that hysterectomy significantly increases the risk of CVD^4,38–40^. This may be explained by the fact that in the present study, we assessed the association between hysterectomy and CHD which is a subset of CVD.

Our results also showed that menopause was significantly associated with CHD after adjustment for age, DM, HTN, and residential area. However, the full multiple logistic regression model showed that this association was not significant after adjustment for most of the confounders. The studies show that postmenopausal decline in estrogen levels has a profound impact on body composition, resulting in altered fat distribution patterns and heightened oxidative stress, which can amplify the risk of metabolic disorders and CHD^41–44^. Consistent with our findings, Dam et al.^45^ investigated the association between menopause and CHD risk in a large cohort of European females and found no significant association after adjustment for confounders. Conversely, Agrinier et al.^12^ et al. studied 1730 French females and found that post-menopausal women have a higher Framingham 10-year risk of CHD compared to pre-menopausal females. However, this discrepancy may be due to different methodologies in both CHD risk assessment and study design.

### Limitations

The reliance on self-reported data for CHD diagnosis, hysterectomy, and TL may introduce recall bias, despite verification via medical records. Self-reporting tends to underestimate particularly in older populations or those with lower health literacy. Second, the absence of data on oophorectomy status is a limitation, as concurrent oophorectomy could confound the association between hysterectomy and CHD. Third, although we adjusted for numerous confounders, residual confounding (e.g., unmeasured lifestyle factors or genetic predispositions) may persist. Fourth, the homogeneity of our sample (Northern Iranian women) may limit applicability to other ethnic or geographic populations, where genetic, cultural, or environmental factors could differentially influence CHD risk.

### Future research directions

Prospective cohorts with repeated measures could clarify temporal relationships between reproductive factors (e.g., hysterectomy timing, menopause onset) and CHD development, while minimizing recall bias. Studies incorporating inflammatory or hormonal biomarkers (e.g., estrogen, CRP) could elucidate mechanistic pathways linking reproductive history to cardiovascular risk. Also, Replication in multiethnic cohorts would help determine whether our null findings generalize beyond Northern Iranian women, particularly in populations with differing genetic or lifestyle risk profiles.

## Conclusion

Our results showed that hysterectomy, menopause, and TL had no significant association with higher odds of CHD after adjustment for confounders.

## Data Availability

The data are available upon reasonable request from the corresponding author.
